# An enquiry concerning the nature of conceptual categories: a case-study on the social dimension of human cognition

**DOI:** 10.3389/fpsyg.2014.00654

**Published:** 2014-06-25

**Authors:** John Stewart

**Affiliations:** Département Technologie et Sciences de l’Homme, Université de Technologie de Compiègne – Cognitive Research and Enaction DesignCompiègne, France

**Keywords:** concept formation, social structures, renaissance philosophy, capitalism, automation

## Abstract

Cognitive Science, in all its guises, has not yet accorded any fundamental importance to the social dimension of human cognition. In order to illustrate the possibilities that have not so far been developed, this article seeks to pursue the idea, first put forward by Durkheim, that the major categories which render conceptual thought possible may actually have a social origin. Durkheim illustrated his thesis, convincingly enough, by examining the societies of Australian aborigines. The aim here is to extend this idea to cover the case of the conceptual categories underpinning modern Western science, as they developed historically first in Ancient Greece, and then at the Renaissance. These major non-empirical concepts include those of abstract Space (Euclidean space, perfectly homogeneous in all its dimensions); abstract Time (conceived as spatially linearized, with the possibility of imaginatively going back and forth); and a number of canonical logical categories (equality, abstract quantity, essential versus accidental properties, the continuous and the discontinuous, the transcendental…). Sohn-Rethel (1978) has proposed that the heart of the conceptual categories in question is to be found in an analysis of the exchange abstraction. This hypothesis will be fleshed out by examining the co-emergence of new social structures and new forms of conceptual thought in the course of historical evolution. This includes the Renaissance, which saw the emergence of both Capitalism and Modern Science; and on the contemporary situation, where the form of social life is dominated by financial speculation which goes together with the advent of automation in the processes of production. It is concluded that Cognitive Science, and in particular the nascent paradigm of Enaction, would do well to broaden its transdisciplinary scope to include the dimensions of sociology and anthropology.

## INTRODUCTION

One of the major merits of Cognitive Science is that it provides a *trans*-disciplinary approach to phenomena that are only too often fragmented into separate disciplines that only communicate on the fringes. Right from the start, the “Computational Theory of Mind” (CTM), whatever its defects and limitations, provides a principled connection between the fields of psychology, neuroscience, and linguistics. However, there is one major discipline in the human sciences that is remarkably absent from the synthesis achieved by cognitive science: and that is sociology. To be sure, there is a whole field which goes by the promising name of “social cognition.” But when one looks closer, it turns out that what is involved is the way that “social factors” can influence or “color” cognition *after the event*; or alternatively, that when both cognition and human society *are already in place*, some cognitive resources can be allocated to thinking about social forms (for example kinship relations, or even explicitly political matters). What is missing is any inkling of the idea that the social dimension may be actually *constitutive* of humanity itself; that a population of individuals who were not *already* profoundly socialized would not be properly human. If this is correct, then the relative weakness of sociology in cognitive science is a fundamental flaw. This critical remark holds for all the currents in contemporary cognitive science. It applies not just to the classical TCM, but also to all the connectionist and neo-connectionist variants, as well as to the nascent alternative of Enaction (Varela et al., 1991; Stewart et al., 2010).

This is clearly a major issue; and it would require at least a whole book to do it anything like justice. In the space of a single article, all I can do is to indicate schematically the existence of the problem; and then to illustrate what may be involved by a single case-study which will be inevitably very limited and partial with respect to the problem as a whole. The specific area I have chosen, in order to attempt a constructive proposal, is that of the genesis of conceptual categories.

## THE NATURE OF CONCEPTUAL CATEGORIES

### A PHILOSOPHICAL PROBLEM: THE GENESIS OF CONCEPTUAL CATEGORIES

As a point of entry into the question I wish to examine, I will base myself primarily on a little-known book by Durkheim (1915). Although this book was published a century ago, it has been virtually ignored. Consequently, the ideas it presents are as new and original as when they first appeared; and I make no apology for taking it as a basic reference. Today, Durkheim is mainly known as one of the founders of modern sociology; but it is worth noting that he had a genuine culture in philosophy. The question of the nature and origin of conceptual categories has indeed a long history in the philosophical tradition; to introduce the question, I will quote directly from Durkheim:

“At the root of all our judgments there are a certain number of essential ideas which dominate all our intellectual life; they are what philosophers since Aristotle have called the categories of the understanding: ideas of time, space, class, number, cause, substance, and so on. These conceptual categories correspond to the most universal properties of things. Thought seems unable to liberate itself from them without destroying itself, for it would appear that we cannot think of objects that are not in time and space, which have no number, and so on. Other ideas are contingent and unsteady; we can conceive of their being unknown to a certain man, a society, or an epoch; but these basic concepts appear to be practically inseparable from the normal working of the human mind. They are like the solid framework which encloses all possible thought.”

(Durkheim, 1915, pp. 21–22)

The next question, then, is this: where do these categories come from? In the philosophical tradition, there are two main answers: *Empiricism* and *Apriorism.* Empiricism is the doctrine according to which the categories are built bottom-up, by bits and pieces, on the basis of regularities in perceptual experience. This viewpoint was developed historically by the British Empiricists: Locke, Berkeley, and Hume. It culminated with Hume’s famous conclusion that the notion of “causality” could only be an illusion (Hume, 1748). The reason is that, however, often we observe that event B follows event A, this can never be a sufficient reason to arrive at the idea that A is a genuine *cause* of B; we can never be sure that next time, A may fail to be followed by B, or that B could occur without *necessarily* being preceded by A. It was this scandalous conclusion, that the concept of “causality” is only an illusion, that provoked Kant to “awake from his dogmatic slumbers,” and lead him to propose his “Copernican revolution” in epistemology (Kant, 1781). Far from experience leading to the categories it was the other around: if there were no categories in the first place, no real experience would exist at all. Kant expressed this by saying that the categories exist *a priori*.

Now the problem here is that these twin doctrines, empiricism and apriorism, are both severely defective. Empiricism is decisively refuted by Kant’s critique; it just does not hold up. On the other hand, if we are looking for a scientific answer to the question of where the categories come from, apriorism is totally inadequate: to say that they exist “a priori” is just putting a name on our ignorance and begging the question. The empiricist answer, saying that the categories derive gradually over time on the basis of empirical experience, is not valid; but empiricism, for all its faults, does at least attempt to give an answer, whereas apriorism just eludes the question altogether. It is arguably because each of these twin doctrines is about equally defective that the philosophical debate between them has been going on for centuries, and would seem to be interminable.

It is important to recognize here that relatively recent developments in cognitive science – in particular the currents of embodied cognition, extended cognition and distributed cognition – represent a significant advance with respect to the “stand-off” between empiricism and apriorism as diagnosed by Durkheim. “Extended cognition” involves recognizing the role of technical artifacts and technological systems in establishing specifically human cognition (Stiegler, 1998; Havelange et al., 2003), and this opens up one route to recognizing the importance of the social domain. The current of “distributed cognition” attributes an important role to interactions between individuals. The weakness of such approaches, in the present perspective, is that they focus on *interactions*, which presupposes that the individuals between whom such interactions can occur are already fully constituted. They thus fall into the trap of “methodological individualism” which has been roundly criticized by Giddens (1977). In the same vein, Steiner and Stewart (2009) have argued that the term “social” is misused when it is used to refer to a situation where there are merely inter-individual interactions (such as the phrase “social insects” to denote ant colonies). What is missing is a proper focus on the social structures which implement the “social synthesis,” a theme we shall return to below. To sum up, none of these recent developments, in spite of their undoubted interest for cognitive science, have yet attributed a fundamental role to the social domain as such. The nascent paradigm of Enaction, which has already been mentioned, would provide a suitable framework for developing a fuller appreciation of the social dimension of human cognition; this has not yet been done, but this article is meant as a step in this direction.

### A SOCIAL ORIGIN FOR THE CATEGORIES?

It was in this situation, that of an awkward stalemate, that Durkheim (1915) proposed an audacious and radically original hypothesis. In order to introduce his hypothesis that the categories have a *social* origin, Durkheim notes that there are actually two *sorts* of knowledge: on the one hand *empirical knowledge*, which relates directly to the interactions between an individual and his environment^1^; and on the other hand knowledge which is framed in terms of the categories, that are essentially social in nature. “Between these two sorts of knowledge there is all the difference which exists between the individual and the social, and one can no more derive the second from the first than one can deduce society from the individual” (Durkheim, 1915, p. 28). Durkheim concludes his Introduction as follows:

“Thus renovated, the theory of knowledge seems destined to unite the opposing advantages of the two rival theories. It keeps all the essential principles of the apriorists; but at the same time it is inspired by that same positive spirit which the empiricists have striven to satisfy. It leaves the faculty of reason its specific power, but it accounts for it and does so without leaving the world of observable phenomena. It affirms the duality of our intellectual life, but it explains it, and with natural causes. The categories… appear as priceless instruments of thought which the human groups have laboriously forged through the centuries and where they have accumulated the best of their intellectual capital. A complete section of the history of humanity is resumed therein. … This is how it is legitimate to compare the categories with tools^2^; for on its side, a tool is material accumulated capital. There is a close relationship between the three ideas of tool, category and institution.”

(Durkheim, 1915, p. 32)

On the face of it, this would appear to be an attractive proposition. It must be admitted, however, that a century later, Durkheim’s proposal has received very little attention from the academic community. The brute fact is that it has not even been criticized; essentially, it has just been ignored. A possible reason for this, or at least a contributing factor, is that the bulk of Durkheim’s long book is devoted to an analysis of the society of the Australian aborigines. It is therefore important to emphasize that Durkheim’s choice of a terrain to gather empirical evidence in support of his hypothesis was in no way guided by a preference for the bizarre or the exotic, but for clear methodological reasons: “in the study of any natural phenomenon which undergoes evolution, there is an immense advantage in starting with the most primitive^3^ form known.” Durkheim illustrates this precept quite explicitly with the case of living organisms: “Biological evolution has been conceived quite differently ever since it has been known that mono-cellular beings exist…. The discovery of unicellular beings has transformed the current idea of life. Since in these very simple beings, life is reduced to its essential traits, these are less easily misunderstood.” (Durkheim, 1915, pp. 18–19). Similarly: “Primitive civilisations offer privileged cases because they are simple cases. That which is accessory or secondary has not yet come to hide the principal elements. All is reduced to that which is indispensable, to that without which there could be no society. But that which is indispensable is also that which is essential, that is to say, that which we must know before all else... But primitive societies do not merely aid us in disengaging the constituent elements of society; they also have the great advantage that they facilitate the explanation of it. Since the facts there are simpler, the relations between them are more apparent. The reasons with which men account for their acts have not yet been elaborated and denatured by studied reflection; they are nearer and more closely related to the motives which have really determined these acts” (Durkheim, 1915, pp. 18–19). Thus, the reason why Durkheim drew mainly on ethnographic studies of Australian aborigines, with supplementary material from studies of Native Americans, was not “simply for the pleasure of telling the particularities and singularities of a very archaic (society)”; but because he hoped thereby to approach the essential constituent elements of human *society*, and to explain them.

Now Durkheim’s adherence to this methodological principle did indeed bear fruit in the clarity and relative simplicity of his conclusions. It became rapidly apparent that in all these “primitive” societies, there seems to be an anthropological invariant: the very nexus of their social life is provided by *religion*: but a religion which is in large part foreign to all idea of divinity or gods. What is at the root of these religious practices is a distinction between the *profane* and the *sacred.* Durkheim therefore goes on to ask what could be at the root of this distinction. An Empiricist might suggest that the notion of “sacred” could derive from extraordinary and possibly “supernatural” events, such as cosmological rarities, showers of falling stars and the like (the theory called “naturism”); or maybe it derives from the phenomena of dreams (the theory of “animism”). But Durkheim very properly dismisses both of these suggestions: since all these phenomena, naturist or animist, do actually occur in the realm of “natural events,” they *cannot* for the life of them suggest the notion of the “sacred” as *different in kind* from the profane. But, Durkheim continues, there are indeed two different *sorts* of reality with which human beings are confronted. On the one hand, there is the ordinary everyday reality of perceived objects and processes (which corresponds non-problematically to the class of the profane); but on the other, there is indeed a quite different *sort* of reality, which is equally non-negotiable by an individual, and that is… social reality! So Durkheim arrives at the conclusion that the “sacred” is neither more nor less than the form in which “the social” presents itself to the consciousness of individuals in these “primitive” societies. His task then becomes to show that the conceptual categories of time, space, and so on have their natural origins in the *religious* categories by which social life is ordered. He was able to muster an immense amount of empirical data to support this hypothesis.

By means of a very thorough and critical appraisal of the ethnographic literature, Durkheim came to the conclusion that, quite generally, the “elementary form of the religious life” was that known as “totemism.” Each tribe is divided into a certain number of *clans* (usually a dozen or so). Each clan is identified by its emblematic *totem*, which is often but not necessarily a particular species of animal or plant (an additional indication of the sacred nature of animals is given by the cave-paintings at Lascaux and elsewhere – Curtis, 2006). The totem is sacred for members of the clan; it is forbidden for consumption (except possibly under special ritual circumstances). This system is inseparably religious and social, confirming Durkheim’s theory concerning the intimate connection between the two. We now come a crucial point: for the Australian, everything which is in the universe is considered to be a part of the tribe; consequently, just like men, all things known are distributed between the clans (Durkheim, 1915, pp. 166–168, where Durkheim cites some examples). Naturally enough, things which are attributed to the same clan tend to have some similarities; this is particularly clear in the case of the phratries^4^, where there are just two classes. Thus, if the white cockatoo is in one phratry, the black cockatoo will be in the other; and the moon is regrouped with the black cockatoo whereas the sun is with the white cockatoo. However, as Durkheim notes with insistence, “the feeling of resemblances is one thing and the idea of class is another…. The contents cannot furnish the frame into which they fit… This is why the idea of class must not be confused with that of a generic image… The best proof of the distance separating these two notions is that an animal is able to form generic images though ignorant of the art of thinking in classes and species.” (Durkheim, 1915, pp. 171–172). Thus, the very notion of “class”, and of systematically and logically classifying entities into a system of classes, is a clear example of a non-empirical, *a priori* conceptual category. What we see here is that the very first systematic classifications that we meet with in history are “modeled upon the social organization, or rather that they have taken the forms of society as their framework. It is the phratries which have served as classes, and the clans as species.” (Durkheim, 1915, p. 169). One could scarcely ask for a clearer or more direct vindication of Durkheim’s hypothesis that the *a priori* categories have a social origin. Durkheim provides analogous demonstrations for other major categories. We cannot go into the details here, but will have to content ourselves with the barest summary: “it is the rhythm of social life which is at the basis of the category of time; the territory occupied by the society furnished the material for the category of space; it is the collective force which was the prototype of the concept of efficient force, an essential element in the category of causality.” (Durkheim, 1915, p. 488).

Durkheim’s methodological choice of starting with “primitive” societies thus paid clear dividends. It does, however, have one disadvantage: it can leave the impression that for “primitive savages” there may well be a relation between forms of thought and forms of social life; but that when it comes to civilized societies, especially in the modern Western world, this “primitive” stage has been surpassed and there is no longer any such relation. This was not at all Durkheim’s own view; he thought that he was not at the end of the story, but just at the beginning. He remarked: “Attributing social origins to logical thought is not debasing it or diminishing its value or reducing it to nothing more than a system of artificial combinations; on the contrary, it is relating it to a cause which implies it naturally. But this is not saying that the ideas elaborated in this way are at once adequate for their object.” (Durkheim, 1915, p. 493). Thus, Durkheim considered that he had laid the foundations for a whole new research program, consisting of following through the whole evolution of human thought, and of relating this to concomitant changes in the forms of social organization. In the next section, we will attempt to respond to this challenge.

## ABSTRACT THOUGHT AND THE EXCHANGE ABSTRACTION

### THE EXCHANGE ABSTRACTION

The aim of this section is to examine whether there is a plausible social basis for the major categories of modern Western thought: more specifically, for the categories which Kant himself identified as being *a priori*, i.e., not derived from empirical experience.

A necessary prerequisite for this task is to characterize the forms of social life in an appropriate way. To this end, I will introduce here the concept of “social synthesis.” Every human society in which there is some degree of division of labor must necessarily have a mechanism which provides functional answers the following three questions: (i) What are the productive activities which will be performed? (ii) How is the sum of all the work to be performed to be distributed between the members of society: *who* will do what? (iii) How are the fruits of this labor to be divided up amongst the members of society: who will receive what? – It is a question of the viability of any form of social life that there should be a mechanism which provides an effective answer to this question (not necessarily explicitly, but in terms of practical results); in the absence of adequate answers, there will be anarchy and the dissolution of the society. It is worth emphasizing that this question of the “social synthesis” is not merely ancillary; it is absolutely fundamental to the very constitution of human society as such.

Now in very broad terms, one can make a distinction between two major types of mechanism for ensuring the social synthesis, which thereby condition two very different sorts of human society; I will call them “traditional” societies and “market” societies. In the great majority of human societies in the past, the mechanism of social synthesis can be designated by the term “traditional”: there is a definite sort of social order, with a specification of the roles of the various members of society, which is reproduced from generation to generation in an essentially unchanged form. In many cases, this social order comprises institutions of discussion and negotiation: the African palaver can serve as a metonymical example. One can also speak of a “communal” mode of production, where the nature of the productive activities themselves integrate in large measure the distribution of their fruits and, upstream, the corresponding division of labor. We may remark that there is some proximity here with animal communities, where in some cases the differentiation of activities necessary for collective survival can be quite sophisticated. However, since no animals have the capacity for language, there is no animal equivalent to the institution of the palaver type.

By contrast with these “traditional” forms of social organization, there are societies (including our own contemporary society) that we can designate by the term “market” societies. Here, a large part of the social synthesis is neither traditional, nor the object of relatively direct discussions, nor integrated with the productive activities themselves; it is delegated to the mechanisms of a *market economy.* In this case, the social synthesis is achieved by the famous “invisible hand” of Adam Smith, according to the laws of supply and demand which are balanced by the mechanism of *prices.* In other words, the social synthesis is not directly achieved as such; it is, rather, the “emergent” result of a whole series of purely local economic decisions, without there being anywhere a coherent vision or conscious will at the level of the whole. It is important to emphasize that in market societies, characterized by a division of labor, economic exchanges play a fundamental role because they determine the *form* of the social synthesis. The life of each individual depends on the activities of production and consumption; but without the intervention of market exchanges none of these activities would occur. Each economic crisis is an object lesson in the fact that the activities of production and consumption are perturbed precisely to the degree that the functioning of economic exchanges is compromised.

The cornerstone of a fully developed market economy is the social institution of *money.* It is important and interesting to note that the invention of money as such was not immediate. The successive steps in what was a long historical process, involving a considerable investment of collective intelligence, have been carefully documented in the work of Simmel (1900). Briefly, some of the major steps were: gift and counter-gift; direct barter; the appearance of certain commodities which were not quite like the others because they were systematically used as intermediaries in market exchanges (grain is a good example); the use of precious metals; and the first instances of coined money, which mark a first culmination of the process and will serve as the basis for our analysis of the *exchange abstraction.* In what follows, I will base myself essentially on the work of Sohn-Rethel (1978).

Market exchange involves an *abstraction* because it requires a rigorous relation of *mutual exclusion* between *use* and *exchange.* The activities of use on one hand, and activities of exchange on the other, are not simply different; they *must* take place separately, during different and mutually exclusive temporal periods. The reason is that the exchange activity serves the sole purpose of a change in owner, in other words a change in the *purely social* status of the commodities as elements of private property. In order for such a change to take place on the basis of a negotiated agreement, the *material* status of the commodities, their physical condition, must remain unchanged during the whole period of the negotiation – or rather, which is even more relevant here, their material status must be *presumed* to be unchanged. Sohn-Rethel (1978) provides a graphic presentation of this key point:

“There, in the market-place and in shop windows, things stand still. They are under the spell of one activity only; to change owners. They stand there waiting to be sold. While they are there for exchange they are not there for use. A commodity marked out at a definite price, for instance, is looked upon as being frozen to absolute immutability throughout the time during which its price remains unaltered. And the spell does not only bind the doings of man. Even nature herself is supposed to abstain from any ravages in the body of this commodity and to hold her breath, as it were, for the sake of this social business of man. Evidently, even the aspect of non-human nature is affected by the banishment of use from the sphere of exchange.”

(Sohn-Rethel, 1978, p. 25)

The practical activity of exchange does not in itself have any meaning in terms of nature; it is purely social by its constitution and scope. Nevertheless, the transfer of ownership that is negotiated under property laws in no way lacks physical reality itself. Exchange involves the movement of the commodities in time and space from one owner to another, and constitutes events of no less physical reality than the use-activities which it rules out. It is indeed precisely because their physical reality is on a par that these two kinds of activity, exchange and use, are so mutually exclusive. Thus, exchange is an *abstraction* because, while remaining inseparable from use (otherwise no-one would bother to exchange the commodities in question), it quite rigorously excludes it. At the same time, it is a *real* abstraction, because it is a perfectly real event in time and space.

To sum up the argument so far: in market societies, there are two registers of spatio-temporal reality which exist side-by-side, but which mutually exclude each other. This point will be so important for what follows that it will be useful to employ specific terms. In German, the register of “use” is designated by the term “first nature” (*erste Natur*); this register is entirely and substantially material. The register of “exchange” is designated by the term “second nature” (*zweite Natur*); this register is entirely social and, by its constitution, perfectly abstract. The same term “nature” is employed to indicate that these two worlds are endowed with an equal degree of spatio-temporal reality, and that they are inextricably combined in the fabrication of our daily life in a market society.

The strange relation between “first nature” and “second nature” is brought to its peak by the social institution of coined money. Money is an abstract, paradoxical entity: it performs a decisive function in the social synthesis, but *unbeknown* to the actors concerned (we will come back to this point). But even if the “exchange abstraction” is practically never thought of *as such* by economic agents, no animal can begin to understand what money is: it is a register that is solely accessible to human beings^5^. Sohn-Rethel (1978) makes this point in striking fashion, and I will cite him again:

“Take your dog with you to the butcher and watch how much he understands of the goings-on when you purchase your meat. It is a great deal and even includes a keen sense of property which will make him snap at a stranger’s hand daring to come near the meat his master has obtained and which he will be allowed to carry home in his mouth. But when you have to tell him ‘Wait, doggy, I haven’t paid yet!’ his understanding is at an end. The pieces of metal or paper which he watches you hand over, and which carry your scent, he knows, of course; he has seen them before. But their function as money lies outside the animal range. It is not related to our natural or physical being, but comprehensible only in our social interrelations as human beings. It has reality in time and space, has the quality of a real occurrence taking place between me and the butcher and requiring a means of payment of material reality. The meaning of this action registers exclusively in our human minds and yet has definite reality outside it – a social reality, though, sharply contrasting with the material realities accessible to my dog. Here we have the spheres of the “first” and “second nature” which we distinguished earlier side by side, and unmistakably divided.”

(Sohn-Rethel, 1978, p. 45)

Marx says quite explicitly that the exchange abstraction never receives a mental representation as such, since its sole expression resides in the *act* of considering that the value of one commodity is equal to the value of another (Marx, 1867, p. 162). Gold, or silver, or any other material entity which lends to money its instantiation as a visible, palpable body is only a *metaphor* for the exchange value, it is not the abstraction as such. In fact, the material instantiations of money do more to mask than to reveal its veritable “second nature.”

Historically, when commodity exchanges spread, becoming multilateral and involving a wide range of commodities, there was an overwhelming practical need to employ one of these commodities as a *general means* for the exchanges of the others. This new role did not in itself, immediately, confer the commodity in question with an appearance that is different from before; but as a means of exchange, it is invested with the *postulate* that it should undergo no material change as long as it continues to exert that function. It is therefore easy to understand that the choice of a “standard-commodity” will fall on an entity that by virtue of its physical durability, its divisibility and its mobility is relatively conform to the required properties. In this way, the *postulate* of immutability, which has its true source in the abstraction of exchange, quite rapidly acquires the appearance of a *consequence* of its particular properties. The fact that a special “aura” attends this commodity “not like the others” does more to confirm than to refute this misleading appearance.

This confusion reaches a summit when the choice for a “standard-commodity” falls on one of the precious metals. On the occasion of each market transaction, it was necessary not only to weigh the metal, but also to melt it and test for purity; in short, it was necessary to relapse into treating them according to their first nature. And precisely for this reason, they failed in the end to perform their function as a universal means of exchange. This deficiency only found its solution with the invention of *coined money*: this step, which was to have such weighty consequences, was first taken in Ionia around 680 BC. With coined money the preceding relation, where its status as exchange-value was subordinated and masked by its material, first-nature status, was overturned. A piece of coined money is stamped in order to signify that it is to serve as a means of exchange and not as a use-object. Its weight and metallic purity are guaranteed by the emitting authority; thus, if it happens that a coin of money has lost weight through wear and tear, the authority in question will replace it free of charge. Its physical matter has become merely the bearer of a social function^6^. A piece of coined money is an entity which conforms to the *postulates* of the exchange-abstraction; it is *presumed* to be composed of a substance which is absolutely unchanging, on which time has no effect, and which is thus unlike any material substance which actually exists in nature.

### ABSTRACT THOUGHT

We come now to a crucial point. We have characterized the form of social life in societies governed by a market economy in terms of the *exchange abstraction*; is it now possible to identify a corresponding form of thought, and more precisely a corresponding set of “*a priori*” conceptual categories?

Sohn-Rethel (1978) introduces his response to this question with a pleasant thought-experiment. The leading role is played by a philosophically minded Athenian from Classical Greece, who asks himself searching questions about the coins of money in his pocket: “What sort of substance *should* these coins be made of?” As none other than the great Plato emphasized clearly, all material objects existing in the world are perishable, corruptible, and unable to resist the ravages of time; but it seems clear that precisely because of this, ordinary material objects are not properly suitable to the function of *money.* Now Plato also speaks of entities of another sort, which are spotless, eternal, perfectly pure, and always strictly identical to themselves: he denotes them by the honorary title of “Ideas.” So, our Athenian asks himself, “are coins of money actually pure Ideas?” Worried, he takes hold of the coins in his pocket, and thinks hard: “These coins *are* real things; and they are real not just for me, but for all my fellow citizens who accept them in payment for wares. Might money be immaterial? – what an absurd idea, no coin could properly be money if it did not have material reality.” So he comes to the reassuring conclusion that the substance that his coins are made of is a *real* substance, as real as any other substance existing in time and space. And yet, this substance is quite different from all these other ordinary substances, because this one is just as immutable as the entities that Plato speaks of. But how can a substance which is immune to the ravages of time exist in time? Nowhere in the whole of nature, and nowhere within the limits of sensory perception, can any such substance be found. But then, how can our Athenian *know* about this extraordinary sort of substance if he cannot see it or hear it or touch it? He knows about it *by thought* and only by thought. Never in all his life has he ever come across this sort of entity, something which is obstinately and uncompromisingly real and yet which is detached from any of the sensory qualities by which things are usually real for us.

This reflection can introduce us to more detailed examination of the formal analogies which exist between the conceptual categories of philosophical thought on one hand, and the distinctive features of the exchange abstraction on the other. It is important to emphasize here that what characterizes each and every one of these conceptual categories is their “canonically apodictic” nature: quite generically, each of them has the remarkable property that once they are identified, in their ideality, it appears intrinsically manifest that they could not be other than they are. At the same time, they are radically non-empirical: there is nothing in our daily experience of nature which is sufficiently similar for it to be at the origin of the concept. What Sohn-Rethel (1978) is suggesting is that actually, there is something in our daily experience that does fit the bill: however, this is not any sort of material reality, but *social* reality.

We therefore hold the germ of an understanding as to how it can be that certain particularities of the exchange abstraction – which is a social form *par excellence* – can be at the root of conceptual categories which are both radically non-empirical, and which can yet be applied to think about material, physical reality. This may be a good place to remark that the relation between social forms and forms of thought, as it is manifesting itself here, is not simple; it is not a question of direct linear causation in one direction or the other. The social forms and the thought forms come about *together*; while there is a sense in which it is the social forms which provide the ground for the conceptual forms (Sohn-Rethel’s presentation can be read in this way), it is surely at least as much the other way round: the cognitive capacity to think in a certain way is a condition for the corresponding form of social life to arise. It is salutary to recall here, as Simmel (1900) has so clearly shown, that the emergence of societies based on a market economy occurred only very gradually, over the course of many centuries. One of the reasons for this is surely that human mentalities had to change in order for this evolution in social forms to be possible.

With this, we have set up the case for supposing that the exchange abstraction may indeed be at the root of the basic conceptual categories of Western thought. It now remains to flesh out this account by developing it in more detail. In the next section, we shall do this in two ways; firstly, by looking at a set of fine-grained “homologies”; and secondly, by looking at the correspondences between social and conceptual forms as they have co-evolved in the course of human history.

## FLESHING OUT THE RELATION BETWEEN SOCIAL AND CONCEPTUAL FORMS

### CHARACTERISTICS OF THE EXCHANGE ABSTRACTION IN ANCIENT GREECE: THE HOMOLOGIES

The account we have given of the correspondence between the exchange abstraction and the abstract categories of Platonic thought have so far been expressed in rather general terms. If this relation is real, it should be possible to spell it out in more detailed terms. Sohn-Rethel (1978) has risen to this challenge, and we shall now present the set of homologies between seven of the canonical set of basic categories, for which he has found corresponding aspects of the exchange abstraction.

#### Solipsism

The doctrine according to which “I alone exist” (*solus ipse*) is a leading leitmotiv of Western philosophy. This doctrine reached the summits with Descartes and Berkeley. In Descartes’ famous “*Cogito ergo sum,*” the “self” in question guarantees its *own* existence – the very idea would collapse if the “existence” in question extended to anything other than the subject of the *cogito*. Berkeley deliberately pushes this solipsism to a provocative limit with his “*Esse est percipi*”: “to be” is neither more nor less than being perceived. In other words, it is not only other subjects but the whole world which only exists to the extent that *I* perceive it. With his usual clarity, Kant summarizes the apodictic character of solipsism: “there is no foundation in theoretical reason which makes it possible to infer the existence of another subject.” This is of course an affront to common sense: in ordinary everyday life, no-one seriously doubts for a moment the existence of other subjects, nor the real existence of the external world. So where could this preposterous idea, which is clearly non-empirical, have come from? There is undeniably a certain irony in looking for an origin in the social domain, because solipsism would seem to be the very antithesis of sociability. But Sohn-Rethel (1978) rises to the occasion.

Since solipsism is a private thought *par excellence*, the first idea that comes to mind concerning the social sphere is that of *private property*. This is all the more plausible in that at first sight it would seem that the institutional principle of private property is logically prior to market exchanges. But Sohn-Rethel (1978) argues that actually the relation is the other way around: the principle of “private property” is actually only a retrospective conceptualisation of necessities that are already inherent in the social *act* of exchange. Let us look at this more closely.

During the whole duration of an exchange transaction, the commodity in question must imperatively be withdrawn from the sphere of use. This is what we have already analyzed above, where we noted that market exchanges induce a rigorous mutual exclusion between *use* and *exchange.* We now have to pursue this analysis, by examining the consequences of this separation for the consciousness of the agents. To do this, we will successively examine the two aspects: first that of use, then that of exchange.

– Concerning *use*, we may note that the minds of the participants in the market transaction are each necessarily engaged with what they are planning to do with the merchandise once they have acquired it, otherwise the motivation for engaging in the transaction would disappear and the exchange would have no reason to take place. It is for this reason that an exchange is an *abstraction* which, in the last resort, is inseparable from use. But we may also note that these thoughts are essentially private: the specific content that each partner has in mind (whether one wishes to acquire some sodium chlorate for gardening, or to make a home-made bomb, for example) does not enter into the exchange as such.

– Concerning now the *exchange*, we may note that it is an action, and that this action is social; but that it is not thought of as such by the agents. In a commodity exchange, whatever the agents think about it (and even if they are not thinking about anything at all other than their private motivations), two principles are *tacitly* implied: (i) that of a mutual exclusion of property (what belongs to A does not belong to B, and vice versa); (ii) the fact of obtaining one object and giving up another does not result from a direct, “natural” action (for example, as in theft), but from an exchange involving mutual consent. In other words, the direct relation to nature is suspended, and replaced by a social relation.

To sum up: what the owners of commodities *do* in the context of a commodity exchange is effectively equivalent to practical solipsism; and this is the case, quite independently of what the agents concerned may or may not actually think or say about it. There is thus indeed a telling correspondence between “solipsism” as a philosophical category, and certain aspects of the exchange abstraction.

#### The unicity of that which is

The first thinker in human history who attained the sphere of “pure thought”, a style of thought quite different from anything that exists in traditional communal societies, was Parmenides (Cornford, 1939). His central concept is designated, in Greek, by the words τo𝜀oν, which is generally translated as “the One; that which is.” This entity is intrinsically and perpetually unchanging; it occupies the whole of space; it lacks all the attributes of sensory perception; it is strictly homogeneous and uniform; it is indivisible; it is incapable of any sort of becoming or decaying; and it is forever immobile. Parmenides emphasizes that the reality and the being of this entity are such that it is intrinsically and literally inconceivable to think that it does not exist. This reasoning is central to his whole doctrine; and it marks the first time in the whole of human history that a conclusion is based on purely logical arguments. Thus, the τo𝜀oν is the starting-point for a thought-process which proceeds by pure reasoning. In other words, what characterizes this style of thought, quite unprecedented at that time, is the fact that this purely conceptual thought grasps the dialectics of truth and non-truth according to the canons of logical *necessity* which is absolutely binding. Parmenides writes: “The fact of thinking, and the thought “it is,” are one and the same thing. For you will never find any thought divorced from that which is, from what the thought is about. For there is not, and there never will be, any thing other than that which is.” Hegel (1833) was later to recognize himself perfectly in this stance, and comments: “This is indeed the fundamental idea. Parmenides marks the beginning of philosophy.”

We may note that the concept of τo𝜀oν is a *premise* for the logical arguments of Parmenides; but the origin of the concept itself is enigmatic. One thing is clear at any rate: it is a radically *non-empirical* concept. It is indeed totally evident that no-one has ever seen (or heard, or touched, or tasted, or smelt) anything at all which bears the least resemblance to this τo𝜀oν. In this respect, it is worth noting that neither Parmenides, nor any of the other founders of Greek philosophy, claim to have personally *invented* their key concepts themselves. Parmenides never suggests, for example, that he arrived at this concept by a process of generalization on the basis of multiple cases in order to arrive at the level of a universal concept. The abstractions which underlie these concepts are of a quite different sort: one *finds* them already there, complete in themselves, totally without any process by which they could be derived. They come from elsewhere, outside and independently of any human thought.

It is in this difficult situation that Sohn-Rethel (1978) proposes his audacious solution to the problem. According to him, the concept of Parmenides corresponds in quite exemplary fashion to a description of the abstract substance from which, ideally, *money* should be made. A market commodity can be exchanged between two private owners precisely to the extent that it has the capacity to be constituted as the object of a mutual exclusion of ownership. It is this capacity which makes it impossible for such a commodity to belong simultaneously to two different owners: a commodity is essentially *one* in the context of a rivalry between *two* owners.

What, precisely, does this “unicity” consist of? It has nothing to do with the indivisibility of the commodity considered as a material entity; it has nothing to do with its actual natural properties. In fact, what is brought into play is not the unicity of the commodities themselves, but the unicity of their *existence.* The ways in which a commodity can be perceived – as an object and in terms of its possible use-value – are as diverse as the persons who perceive it; but it *exists* in a single world which is common to all the private individuals, and this is the world of market exchanges.

The unicity of the exchange abstraction is thus absolutely fundamental, because it is this unicity which constitutes it as an instrument capable of realizing the social synthesis; in other words, of conferring on the society in question its coherence and its unity. There is thus an astounding formal concordance between this unicity of the exchange abstraction, and the ontological unicity of the τo𝜀oν of Parmenides which is the founding abstraction of philosophical thought.

#### Abstract quantity

The work of the formalist school of mathematics (Weir, 2011), notably following Hilbert, have made quite explicit something which was up until then merely implicit in the whole of “pure mathematics”: this is the perfectly *abstract* quality of “natural numbers.” The mathematical definition of these numbers involves a notion of “abstract quantity” defined by nothing other than the relation “larger than” (>), “less than” (<), or “equal to” (=)^7^. The fact that the very considerable work of the formalist school was necessary to make these concepts explicit is an eloquent indication of their abstract, non-empirical nature. “Numbers” as we experience them empirically are not at all built in this way (which explains the abstruse, non-intuitive nature of “formal mathematics” which has given such headaches to pupils and teachers alike in schools where a well-intentioned but possibly quite misguided attempt has been made to introduce this new program of “modern maths”). Numbers as we come across them in daily life are never separated from the objects that are to be counted; what we can actually experience empirically are twenty sea-shells, or twenty cows. But then, if the concept of “pure quantity” cannot be derived from empirical experience, where on earth could it have come from?

Sohn-Rethel (1978), continuing his analysis of the exchange abstraction, sees an answer to this enigma in the following way. The act of exchange contains within itself the *postulate* that the two sets of commodities to be exchanged are *equal*. But how are we to define and to characterize this “equality”? It does not reside in the identity of the commodities, because if they were completely identical there would be no point in exchanging them; only *different* commodities are exchanged. Neither are the commodities considered to be equal in the minds of the agents, because their action would become absurd if they did not see any advantage in realizing the exchange. What is more, this sort of evaluation only exists in the solipsistic register of each individual conscience; from one person to another, such evaluations are not comparable. Nevertheless, it is of the very essence of the postulate of equality that it transcends the gulf of experience between the agents. The postulate of equality does not derive from their experience; the only thing they to agree is that the two sets of commodities can be exchanged. The two sets of commodities are *rendered equal* by the very act of exchange; they are not exchanged in virtue of any sort of “equality” that they possess in themselves.

An act of exchange of this sort, which ends up by *postulating* the equality of the sets of commodities, may well be preceded by a negotiation, by a sort of petty bargaining where what is at stake for each agent is “take more” and “give less.” Now it is true that many commodities can be measured in dimensional units (tons, gallons, square metres, and so on). But the comparative terms “more” and “less” employed during the bargaining do not involve a quantitative comparison between, for example, tons of coal, gallons of petrol, or square yards of fabric. The relational equation postulated by an act of exchange leaves behind it all such dimensional measure, and *establishes* a level of pure non-dimensional quantity. At the end of all this we find, very precisely, the level of pure numbers defined by nothing other than “>,” “<,” and “=.”

#### Abstract time and space

In the list of categories of synthetic *a priori* judgement, as Kant set them out, an important place is occupied by the concepts of time and space. This space is that of Euclidean geometry: it is notably characterized by the fact of being rigorously homogeneous and isotropic. As Jaynes (1976) has pointed out with great perspicacity, time is only accessible to reflexive consciousness, and indeed to scientific thought, if it is metaphorically transposed to this conceptual framework of an ideal space: in this context, “time” is nothing other than a Euclidean point which advances uniformly along a straight line which is also Euclidean. It may not be necessary to dwell at length on the totally non-empirical nature of these concepts, since this thematic leitmotiv is becoming familiar. The space in which we move in the course of our daily life is anything but homogeneous and isotropic (Merleau-Ponty, 1945). As embodied beings, we are constantly subject to the anisotropic influence of gravity (in fact even this characterization is already idealized with respect to our phenomenologically immediate lived experience). And even the space of our movements in the two horizontal dimensions is not homogeneous, being encumbered in all sorts of ways. We have no perception of spatiality outside our actions (this is particularly clear in the “enactive” approach to cognition and perception). Now these actions are constitutively dependent on the particularities of our embodiment and of our natural *Umwelt*; and both of these are anything but homogeneous and isotropic. And as for time, considered as we have immediate lived experience of it, its “framing” by the metaphor of spatiality is in no way empirically given; and on the other hand, it is characterized by biological and psychological rhythms, day and night, which once again are anything but homogeneous and linear. So where could the rigorous *ideality* of the Euclidean conceptions come from?

As we may expect, Sohn-Rethel (1978) sees the source of this ideal abstraction in the switch which comes when the categories of space are applied not at the level of use, but at the level of market exchanges. At the level of use, which we interpret here as covering the totality of all human activities in relation with nature, space, and time are inextricably linked to natural events and human activities: as for example in the ripening of harvests, the seasons of the year, hunting animals, the birth, and death of human beings, and generally everything that happens in the course of life. Now every act of exchange requires abstracting away from all this, because the commodities are supposed to be quite immutable during the whole duration of the exchange. The transaction does take a certain lapse of time, because one must include the delivery of the commodities and the payment which concludes the exchange. But the totality of this time is emptied of all the material realities which make up its content at the level of use.

Very similar considerations apply to space, for example the distance that the commodities must cover when they change owners. While the commodities are in transit from the old to the new owner, the equality between the two sets of commodities holds at each position and at each instant *in exactly the same manner* as at any other position and time. It is for this reason that time and space, when they are applied to the exchange,* must* be perfectly homogeneous. They are also continuous, in the sense that they allow for an interruption at any moment during the transit. In other words, the exchange abstraction excludes everything which makes up history, whether it be human history or natural history. The empirical reality of facts and events, and their descriptions which make it possible to differentiate one local time and position with respect to another, is entirely obliterated. This is how time and space acquire that character of universality and atemporality which *must* mark the exchange abstraction in each of its traits.

#### Substance and accidents

It is well known that Aristotelian logic operates a fundamental distinction between the “essential” properties of an object – in brief, the necessary and sufficient properties for an object to belong to a certain class of objects (for example, being “a tree,” “a cat,” and so on) – and the “accidental” or contingent properties, those that an object can have (or not) without affecting its membership of a class (for example, the fact that a cat is gray or ginger). In its more highly developed form, this distinction becomes that between “primary” properties – in physics, these reduce essentially to the mass, the position and the state of movement of a particle – and “secondary” properties such as its color, its sound, its smell and so on. It is pretty evident that this conceptual scheme – which gives pride of place, need it be said, to the “essential” or “primary” properties – is the exact opposite of the empirical situation, for everything that can actually be perceived is relegated to the status of “accidental” or “secondary” properties. But if the “essential,” “primary” properties are non-empirical, where do they come from?

Sohn-Rethel (1978) once again finds an answer in the exchange abstraction. In fact, we have already largely presented what is at stake: the “ideal” substance of which money should, ideally, be made is very precisely devoid of all sensory qualities; all that remains are the properties necessary for it to transit in abstract space and time. Let us recall, once again that we are dealing with an “abstraction” precisely because the *use*-value of a commodity (and without which it would actually not have any exchange-value either) is constituted precisely by its empirical qualities.

#### The continuous and the discontinuous

One of the grand themes which characterize the whole tradition of Western mathematics is the tense opposition between the continuous and the discrete (Salanskis, 1992). Already in ancient Greece, this gave rise to the paradoxes of Zeno – Achilles who would arguably never quite catch up with the tortoise. Another key moment was the invention of differential calculus by Leibniz and Newton. Once again, this is a concept that does not arise in the empirical sphere of daily practice; and once again, Sohn-Rethel (1978) finds roots for it in the exchange abstraction. On the basis of what we have already said, and summing up, it is clear that an act of exchange *must*, intrinsically, be described as *the abstract movement, in abstract space and time (i.e., homogeneous, continuous and empty) of abstract substances (materially real but devoid of any sensory qualities) which do not undergo any material change and which can only be differentiated in a quantitative and non-dimensional manner.* Now on one hand the constancy of the exchange value confers a continuity to the whole process of exchange; but on the other, it must be possible to *interrupt* the movement of the commodities at any place and time in order to verify the constancy of their value, and this cuts their movement up into a number of discrete packets. This contradictory nature, both continuous and discrete, comes from the social origin of their abstract nature.

#### The transcendental

A final element in this list resides in the feature that above and beyond the relatively fine and specific details of the homologies we have examined in a–f, there is an over-riding, generic characteristic of the conceptual categories. Although philosophers are general silent (not to say evasive) concerning the genetic origin of the Kantian categories, they all agree that these categories are both “given” *a priori* in a non-empirical fashion, and at the same time absolutely compelling in their apodictic normativity. “Logic” in this sense has the property that it could not be other than what it is. This is the meaning of the philosophical term “transcendental.” But where could this remarkable property come from? Once again, we find a corresponding characteristic on the side of the exchange abstraction. This abstraction is indeed founded not on empirical facts, but on *social postulates*; and there is a sense in which they postulates could not be other than they are, on pain of the entire edifice collapsing (and in this case, in the framework of a market society, all activities of production and consumption would cease and the whole society would materially collapse). We can make an impressive list of these postulates which all have in common this feature that on the one hand they are *pure postulates*, but at the same time endowed with a sort of intrinsic necessity. Thus: it is a *postulate* that the use of commodities should be suspended until the action of exchange is completed; that no modification should occur in the physical state of the commodities, and that this postulate must be maintained even if empirical facts would seem to run counter to it; that the commodities which are exchanged should count as equivalent in spite of all their manifest empirical differences; that the fact of acquiring and giving up commodities is bound to *a priori* conditions concerning their exchangeability; that commodities change owners by transiting from one place to another without being materially affected, and that this movement occurs in an “empty” space. None of these formal concepts invokes any sort of empirical, factual observation; they are all *norms* that the exchange of market commodities *must* satisfy in order to implement the social synthesis.

#### Conclusion

Having examined in some detail several of the “homologies” identified by Sohn-Rethel, this may be the place to pause and to pose anew the question of the *status* of these homologies; in other words, the *nature* of the putative relation between forms of social life and forms of thought. Quite generally, if there is a correlation between two entities X and Y, and this correlation is not merely an illusion due to pure chance, there can be three reasons for this. It may be that variation in X is a cause of variation in Y; or that variation in Y is a cause of variation in X; or yet again that there is a common cause of variation in both X and Y (of course these three possibilities are not necessarily mutually exclusive). In the present case, the initial formulation of the question by Durkheim tended to suggest that there was a causal relation in the direction from social forms to conceptual forms. But we have already suggested, at the end of section 3, that the relation almost certainly functions also in the other direction: the cognitive capacity to think in a certain way is a condition for the corresponding form of social life to arise. And finally, reflection on the nature of the “homologies” proposed by Sohn-Rethel (1978) raises a third possibility. We have emphasized, concerning the τo𝜀oν of Parmenides, the apodictic nature of the concept; it seems to have a sort of inner necessity, such that it could not be other than it is. And concerning the category of the “transcendental,” we have again noted this apodictic quality that has so impressed philosophers over the centuries; but we suggested there that this striking quality is in resonance with the fact that the various aspects of the exchange abstraction are also *pure postulates* with a sort of intrinsic necessity. In other words, the social forms and the conceptual forms we have been examining have a fundamental feature in common. To sum up, it would seem that all three of the possible reasons for a correlation between social forms and conceptual forms make a significant contribution.

### HISTORICAL EVOLUTION

The “homologies” that we have examined in the previous section are based on the state of affairs at a particular point in time and place. This situation – that of Ancient Greece – is indeed a key moment; but it is nevertheless only one moment in a continuous and ongoing process. The relationship between forms of thought and forms of social life comes into fresh light if we look at their *co-evolution* in the course of human history.

If we take as a starting-point our pre-hominid ancestors, the totemic systems of the aborigine societies that we examined in section 2 already represent a first appearance of a form that is both social and conceptual.

The second major step is one that we have also seen already: the identification of the major conceptual categories of Western thought by the Ancient Greeks. What is nevertheless worth remarking here is the amazing historical coincidence of time and place: Athens, around 400 BC, saw both the work of Plato and Aristotle, and the inversion of coined money. An additional factor, which is of both social and cognitive importance, is that this was also the epoch of the invention of alphabetic writing (see Goody, 1977 for a fuller exposition of the significance of this momentous event, which marked the entry into history in the modern sense of the term).

The third step is that of the European Renaissance, in the 16th and 17th centuries. As its name implies, this was a period of a return to the high intellectual ideals of Ancient Greece, after the decline of the Roman Empire and the interlude of the “Dark Ages.” It was more than just a return, however; since this was also the period of the birth of “Modern Science.” What is to be noted here is that the Greeks invented almost all the concepts under the sun; but they did not really put them to use to discover new, fundamental knowledge. In a sense, the Greek concepts were strangely static; it is almost as though their canonical nature, their apodictic character which the Greeks themselves thematized explicitly, left them bereft of the possibility of development. The spirit of modern science contrasts with this: scientific concepts are eminently put to use to create unprecedented knowledge; and because they are used in this way, they themselves evolve.

Is there a corresponding difference in the character of the exchange abstraction? If we look for it, the answer is yes. For the Greeks, money was used essentially as a means of external commercial exchanges (Scheidel et al., 2007). Domestic production was not involved; to put it bluntly, this is because it was performed by slaves who were not paid money for their work. This is precisely what changed at the Renaissance, which was also the period of the invention of Capitalism. The key point is that now, money was invested in the production process itself, with the invention of salaried labor. And need it be pointed out that Capitalism is intrinsically *dynamic*: the capital invested is returned with a profit, which can then be reinvested and so on, leading to a potentially exponential growth. This fits remarkably with the fact that at the heart of modern science, most notably with Newton, there is the concept of a *dynamic system*; or more precisely, a State-Determined Dynamic System (SDDS; Aubin and Dalmedico, 2002). There is probably no better way of illustrating the fecundity of our hypothesis of a profound link between conceptual forms and social forms, than to use it in a back-and-forth fashion to sharpen our identifications of both sides of the relation. What then can the concept of a SDDS point to in the functioning of Capitalism?

An important feature is that a SDDS is perfectly “autonomous”: once it is set up, and the dynamic law governing the temporal evolution of its “state” is specified, everything thenceforth occurs without the least “external” intervention. Laplace, who emphasized the radical determinism of such a system, is reputed to have replied to Napoleon when the latter questioned him about the place of God in his system: “Sire, I have no need of that hypothesis” (Rouse Ball, 1908). This can point us to the fact that in a truly capitalist system, the process of production is *theoretically* automatic. It is true that it is common to speak of a capitalist of this sort as a “manufacturer” – as though Mr Ford, for example, had really made thousands and thousands of cars with his own hands; but this is misleading. How does the capitalist fulfill his role as a “producer”? He does not accomplish this by his own work: he achieves it neither with his hands, nor with tools and machines that he would operate himself. He achieves it by means of the money he has invested as capital, *and with nothing else*. “The process of work is a process between entities that the capitalist has bought,” says Marx (1867), “entities that belong to him.” In fact, if ever a capitalist did come to lend a hand himself, that would only show that he had partially failed in this role as a capitalist entrepreneur, and strictly speaking he should pay himself a salary for that manual work. In other words, the role of “producer” falls on an entity which does not perform a single productive function in the work-process. To sum up the essential point: the key characteristic of the production process, from the point of view of the capitalist entrepreneur who invests in it, is that this process should function *all by itself.* The power of the capitalist system resides in this *postulate* of the self-acting or “automatic” nature of the production process.

It is important to note that a postulate of this sort does not necessarily correspond to a historical reality; in fact, as we shall see below, it will require centuries before the social reality of production relations began very progressively to assume the ideal form that we have just described; and even then they did so imperfectly. This only makes it clearer than ever that the postulate of the automaticity of production processes does not come from any empirical source in the actual technology of production; it is rather the other way round, the fact that in the course of the historical evolution of technology the latter progressively comes to conform to the “ideal” in question is a *consequence* rather than a cause of this postulate. The postulate itself is in no way empirical; it is clearly in the realm of the non-empirical *a priori*; and what we have seen is that it is *formally intrinsic* to the social relations of production in a capitalist society. The formal homology between this *postulate*, which is social through and through, and the Newtonian concept of a SDDS which is also based on a *postulate*, is quite impressive.

Finally, we come to the contemporary period. The lead here is given by the idea that we have just expressed: theoretically, from the point of view of a capitalist, profits should ensue *automatically*. Now it may be thought that this idea is a little far-fetched; in a small start-up enterprise, the budding capitalist is likely to do quite a bit of the work himself; and even later, when the enterprise has grown, he still has to do a lot of real work – buying the raw materials, setting up the factory, and equipping it with all the necessary tools, hiring the salaried workers and putting them to work, ensuring that their salary demands will not become excessive – and even then he has not finished, because he must take care of marketing the products once they have been made. But the fact is that history has taken care of bringing to light the kernel of truth in this theoretical idea: over the last century, the core of capitalism has shifted away from entrepreneurial capitalism to the financial sector. Today shareholders, and at a larger scale the great financial corporations, indeed do little else than accumulate the profits and reinvest them on the financial markets; thus coming remarkably close to the theoretical ideal.

So much for the “social relations” side of the picture. What about the “cognitive” side? Here again, it is the theme of “automaticity” that provides the insight. The hallmark of the contemporary scene is the digital computer, which is playing an ever-increasing role. It may not be necessary to labor the point: the essential feature of a computer is that operations on formal symbols are carried out *automatically* and, thanks to electronic technology, with ever greater speed and capacity. The full significance of this comes from the fact that this automaticity is not restricted to merely abstract operations, but that it is being linked to real material production. A clear indication of this is the ever-increasing importance of robots in the production-line; at a deeper level, it is important and fascinating to realize that what has made this link-up possible and effective is the wealth of *scientific knowledge* that has been accumulated since the Renaissance. It is indeed modern science that provides the link between conceptual forms on one hand, and the possibility of effective material action on the other.

An important consequence of this automatization of the production process is that the social institution of salaried employment is coming under pressure. Mass unemployment is of course a social scourge, and is morally unacceptable. However, it is equally clear that as production processes tend towards complete automation, there will be less and less materially productive work to go round. The question is whether it is appropriate to resist this trend; or whether any such attempt is akin to King Canute ordering the sea-tide not to advance, and thus doomed in advance. This is a political question, to which we will return in conclusion.

## GENERAL CONCLUSION

A century after Durkheim made the audacious suggestion that the fundamental conceptual categories may have an origin in the forms of social life, this hypothesis has still not received serious attention by the academic community. Concomitantly, and this is in all probability this is not an accident, the question of the origin of the conceptual categories (when it is not simply eluded) is still in the state of wavering between the twin alternatives of apriorism and empiricism which remain equally unsatisfactory for the very reasons so clearly exposed by Durkheim. It is difficult to avoid the haunting impression that all is not well in the house of Reason.

To sum up the arguments presented in this paper, our final conclusion is that there is indeed a strong relationship between social forms and conceptual forms, including the case of societies governed by a market economy. Indeed, we may go so far as to suggest that social forms and conceptual forms may actually be inseparable; and this for three convergent reasons. Firstly, specific cognitive forms are necessary for the social form to function (more specifically, only humans are able to understand what “money” is – cf the anecdote of the dog at the butcher). Secondly, the evolution of social forms (more specifically, the successive forms of capitalism) *drives* a corresponding evolution in mental forms. Finally, the cognitive forms and social forms in question share a fundamental, constitutive characteristic, that of *abstraction.*

Now if there really is such a strong relationship between the forms of social life and the prevalent forms of thought, a question may well be asked: why is it that this relationship is not more immediately apparent, both to analysts and to members of the societies in question? An attempt to answer to this searching question brings us to the domain of ideology – the particular hallmark of successful ideology being that it does not appear as such. Two illustrations may help make this point. Contemporary capitalism has reached a near-perfect stage^8^, where immense profits ensue to the financial sector for doing practically nothing of any social utility – but these profits seem virtually invisible, both to the tax-collector (the rate of taxation on capital gains is less than that on the salaried earnings of the common worker), and to public consciousness which seems to find this situation quite normal; all this at a time when much is made of the “economic crisis,” and national governments are heavily in debt and held to severe budgetary restrictions. Another illustration of this impressive blindness concerns the situation we have already described, created by the fact that the processes of production are in large part automated. One might have thought that this would open up near-utopian perspectives: if these gains in productivity could be shared in a socially equitable fashion, all members of society would be able to devote the main part of their waking hours to activities that they considered intrinsically rewarding. But instead of that, the very same situation is widely interpreted as a demoralizing threat to salaried employment.

To link these considerations with our previous discussion, we may recall the remark of Marx when he says that the exchange abstraction never receives a mental representation as such, since its sole expression resides in the *act* of considering that the value of one commodity is equal to the value of another. Putting this together with the illustrations of our social ignorance, there need be little surprise that we are largely unaware of the implications of our social situation. The fact that we have no clear consciousness of the effects of our social life on the way we function cognitively cannot be taken as evidence against the hypothesis put forward in this paper. The illustrations also show, however, that this social ignorance has deleterious consequences. Consequently, if the arguments presented here can contribute to even a modest increase in our social awareness, this paper will have been well worthwhile.

Finally, I return to the more general issue addressed in the introduction to this paper: the importance accorded to the social dimension by Cognitive Science. In the space of a single article, it has obviously been out of the question to treat this issue exhaustively. The methodological choice has been made to proceed by metonymy, by concentrating on a case study. The specific domain that has been selected – the origin of conceptual categories – reveals that the social dimension has indeed been systematically ignored. Two major contributions in this area, those of Durkheim and Sohn-Rethel, have received virtually no serious attention by the academic community. Our re-examination of this work has shown that these studies are certainly incomplete, but are basically sound. The conclusion is that there is a call for in-depth follow-up of this question. And enlarging beyond this metonymical example, the social dimension of cognition is worthy of substantial development.

## Conflict of Interest Statement

The author declares that the research was conducted in the absence of any commercial or financial relationships that could be construed as a potential conflict of interest.

## Footnotes

1 Durkheim remarks elsewhere that if a man were reduced to having only empirical knowledge based on individual perceptions of this sort, “he would be indistinguishable from the beasts” (Durkheim, 1915, p. 487).

2 In view of the social importance of tools, and indeed the thesis that “Technology is Anthropologically Constitutive” (Stiegler, 1998; Havelange et al., 2003; Steiner, 2010) it is fascinating to see here that Durkheim himself spontaneously makes the association between conceptual categories, tools and social institutions.

3 This reference, here and later, to “primitive” societies is of course politically incorrect. I nevertheless employ this term (in “scare quotes”) in the same sense of “primordial” that Durkheim uses when referring to single-cell organisms. It should go without saying, but maybe even better by saying it explicitly, that when I do use this term in this text, there is no negative connotation (on the contrary, these societies were arguably far less alienated than our own contemporary society).

4 Although it is not always the case, far from it, certain tribes are organized in just two phratries, with half the clans belonging to each phratry.

5 Chen et al. (2006) have suggested that capuchin monkeys may have some capacity to engage in exchanges with conspecifics. It is actually reassuring that some animals may have an opening in this direction, since this provides a basis for possible evolution towards distinctively human forms of understanding. However, no behavior of this sort is found spontaneously in the wild. It must be re-emphasized that a number of so-called “primitive” human societies (Aborigines and native Americans in their natural state before meeting with Europeans) make no use of money; and, as Simmel (1900) has pointed out so clearly, even in humans the development of a monetary system was a long and very gradual process, spanning centuries. The basic point made is thus remains valid.

6 This also explains how it is that the same function can be performed by simple pieces of paper… as long as they bear inscriptions which cannot be easily forged so that they carry the same guarantee. An anecdote may provide a pleasant illustration of the striking contrast between first and second nature which comes into play with bank-notes. When I was seven years old, I inadvertently left a bank-note in my trouser pocket when it went to be laundered. I was amazed to see my mother recuperate some damp fragments of the note, which still bore in barely legible form the number of the note, whereupon she took them to the bank and obtained in exchange… a brand-new bank-note!

7 I thank one of the reviewers for pointing out that technically, the definition of “natural numbers” also requires the core concept of a “successor function.” Sohn-Rethel does not address of the notion of a “successor function,” nor the passage from “abstract quantity” to “natural number.”

8 “Near-perfect” in its own twisted terms – of course this is anything but “perfect” from a humanistic point of view.
